# Thermal resilience of ensilicated lysozyme *via* calorimetric and *in vivo* analysis†

**DOI:** 10.1039/d0ra06412b

**Published:** 2020-08-12

**Authors:** A. Doekhie, M. N. Slade, L. Cliff, L. Weaver, R. Castaing, J. Paulin, Y.-C. Chen, K. J. Edler, F. Koumanov, K. J. Marchbank, J. M. H. van den Elsen, A. Sartbaeva

**Affiliations:** Department of Chemistry, University of Bath Claverton Down Bath BA2 7AY UK a.doekhie@bath.ac.uk; Material and Chemical Characterisation Facility, University of Bath Claverton Down Bath BA2 7AY UK; The Medical School, Framlington Place, Newcastle University Newcastle upon Tyne NE2 4HH UK; Department for Health, University of Bath Claverton Down Bath BA2 7AY UK; Department of Biology and Biochemistry, University of Bath Claverton Down Bath BA2 7AY UK

## Abstract

Ensilication is a novel method of protein thermal stabilisation using silica. It uses a modified sol–gel process which tailor fits a protective silica shell around the solvent accessible protein surface. This, electrostatically attached, shell has been found to protect the protein against thermal influences and retains its native structure and function after release. Here, we report the calorimetric analysis of an ensilicated model protein, hen egg-white lysozyme (HEWL) under several ensilication conditions. DSC, TGA-DTA-MS, CD, were used to determine unfolding temperatures of native, released and ensilicated lysozyme to verify the thermal resilience of the ensilicated material. Our findings indicate that ensilication protects against thermal fluctuations even at low concentrations of silica used for ensilication. Secondly, the thermal stabilisation is comparable to lyophilisation, and in some cases is even greater than lyophilisation. Additionally, we performed a mouse *in vivo* study using lysozyme to demonstrate the antigenic retention over long-term storage. The results suggest that protein is confined within the ensilicated material, and thus is unable to unfold and denature but is still functional after long-term storage.

## Abstract 2

Ensilication is a novel method of protein thermal stabilisation using silica. It uses a modified sol–gel process which tailor fits a protective silica shell around the protein to enable room temperature storage of biopharmaceuticals.

## Graphical abstract

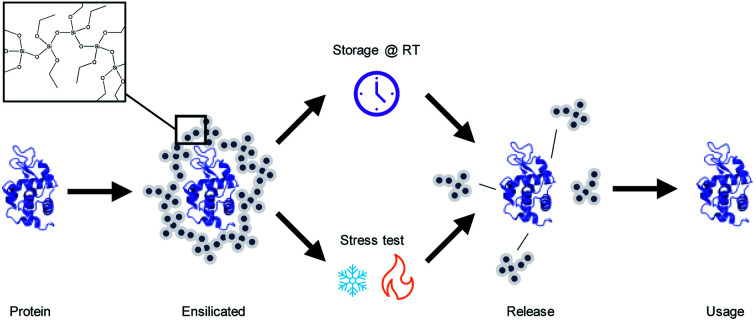

## 1 Introduction

Protein functionality occurs as a direct result of intramolecular interactions between amino acid side chains of polypeptides.^1^ This causes the protein to fold into a specific three-dimensional conformation which is adapted to function efficiently at the protein's natural environmental temperature. Proteins are susceptible to degradation by a number of environmental factors, including changes in pH and temperature.^1^ These changes can alter the tertiary structure leading to protein unfolding, aggregation and precipitation in solution.^2,3^ Previous strategies employed to increase protein stability in solution included excipients, buffer compositions, ionic liquids and encapsulation.^4,5^ Lyophilisation, freeze-drying, is the current gold standard in biomolecule preservation.^6,7^

Ultimately, this study aims to further support the future application of ensilication as a novel approach to biopharmaceutical stabilisation.^8–10^ In order to remain biologically active, the majority of protein-based biopharmaceuticals must be stored and distributed between 2–8 °C in what is referred to as the ‘cold chain’. Changes in temperature outside this range lead to inactivation due to cold chain failures that can often be observed in low-income countries.^8,11–18^ Some of these necessary biopharmaceuticals, such as vaccines, contain excipients which renders them unsuitable for lyophilisation. Other bioactive compounds lose their functional integrity upon lyophilization without additional stabilisers.^19–21^

Therefore, we propose ensilication as an alternative specifically to these because it does not involve freeze/vacuum drying, but rather a softer approach *via* electrostatic attachment and room temperature drying.^8^

In contrast with conventional protein encapsulation, where the particles (for example, PLA/PLGA^22^) are solidified and stored in liquid, our method of ensilication applied in this study, using tetra-ethyl orthosilicate (TEOS) as a precursor, which creates a protein loaded dry silica powder.^8^ Applying this methodology with lysozyme, we found that native protein structure and function were retained following ensilication in our proof of concept study.^8^

Here, we utilise calorimetric methods, to investigate the thermal resilience of this ensilicated lysozyme. Differential scanning calorimetry (DSC) is a physicochemical method that can determine phase transitions in solid or liquid matter by measuring changes in heat flow. This technique has previously been used to investigate thermodynamic properties associated with protein stability and unfolding.^23–27^ This enables us to evaluate where the thermal midpoint (*T*_m_) of transition presents itself. At this specific temperature, the vibrational energy will disrupt the native conformation causing the protein to denature, thereby losing its tertiary structure.^28–30^ Use of this approach allows assessment of ensilicated protein in powder and native/released protein in solution, indicating whether ensilicated protein has the mobility to unfold under heating/freezing within its silica shell. Furthermore, varying the silica ratios used for ensilication would indicate the optimised silica to protein ratio required to render a protein thermally stabilised while allowing for the easiest possible release of the protein from the silica shell.

DSC combined with the measurement of weight loss over heating (thermogravimetric analysis, TGA) and associated elemental analysis (mass spectrometry, MS) allows us to demonstrate the comparison between lyophilised and ensilicated lysozyme. Complimentary to this is the use of circular dichroism (CD) to measure the protein unfolding.^31^ Enzymatic analysis of ensilicated material exposed to heating/freezing allows investigation into the retention of catalytic activity between lyophilised (reconstituted) and ensilicated (released) lysozyme. The methodologies will provide evidence on the thermal resilience of ensilicated lysozyme from a calorimetric perspective.

Our initial study demonstrated the proof-of-principle for ensilication with several biological entities.^8^ Lysozyme, horse haemoglobin and tetanus toxin C fragment were model proteins on to which ensilication was applied. The research demonstrated structural retention of these at protein primary, secondary and tertiary level. Functional capacity using enzymatic or antigenic assays confirmed the applicability of ensilication. The methodology in that study was focused on short term exposure of ensilicated material to heating. Several aspects of the ensilicated thermal resilience were not investigated.

Therefore, in this study, we investigated the thermal resilience of ensilicated lysozyme, used as a reference model. We assessed the impact of various silica ratios used during ensilication. Then analysed the effect of freeze-thawing on the function capacity of ensilicated-then-released lysozyme. Finally, we verified the antigenic ability of non-desiccated ensilicated lysozyme stored up to 3 years at room temperature, compared to a month old sample and native heated, in an *in vivo* trial. By providing an alternative through ensilication we aim to drastically improve the shelf-life of biopharmaceuticals which results to greater accessibility and availability of these, frequently life-saving, therapeutics.

## 2 Experimental section

### 2.1 Native, ensilicated and released lysozyme

#### 2.1.1 Native lysozyme

Lyophilised hen-egg white lysozyme (Sigma, UK) was reconstituted in phosphate-buffered saline (PBS) at pH 7.2 for calorimetric analysis as native control.

#### 2.1.2 Ensilicated lysozyme

For ensilication, lysozyme was reconstituted in 50 mM pH 7 Tris buffer at 1 mg ml^−1^ and ensilicated at ratios of 1 : 20, 1 : 50 and 1 : 100 after which it was vacuum dried and stored at ambient conditions, 20 °C. Ratios correspond to the volume of pre-hydrolysed TEOS added to bulk reaction volume, *e.g.* 1 part of pre-hydrolysed TEOS to 20 parts of reaction volume containing 1 mg ml^−1^ lysozyme. Hydrolysed TEOS was prepared by mixing 1 : 1 TEOS and ddH_2_O with stirring at 350 rpm. The addition of HCl (1 : 500) catalysed the reaction. Once the TEOS : H_2_O phases became homogenous, the designated amount was added to 100 ml of buffered protein solution. The sediment formed during condensation of protein-silica nanoparticles was collected after 20 minutes for all batches made. From here on we will describe the 1 : 20 ratio as high silica, 1 : 50 as standard and 1 : 100 as low silica ratio.

#### 2.1.3 Released lysozyme

Release of ensilicated material was done according to previously published instructions using acidified Na-F buffer(8). This process solubilised the silica and this was removed by dialysis. The released lysozyme was dialysed into phosphate-buffered saline (PBS) at pH 7.2 for calorimetric analysis using SnakeSkin dialysis tubing (3.5 K MWCO). Protein concentrations were assessed using Pierce™ BCA assay or *via* UV-Vis spectrometric analysis at 280 nm.

#### 2.1.4 Detection of ^29^Si after release

The release mechanism is a sequence of several steps that allow the ensilicated material to be released. Silicon (^29^Si) nuclear magnetic resonance (NMR) spectroscopy was employed to assess the presence of silica species after release and dialysis. Native lysozyme solution was used as non-silica present spectra and compared to released ensilicated lysozyme. A range of +60 to −180 ppm was deemed appropriate for analysis since all silicon containing compounds in the reaction give rise to ^29^Si NMR peaks within these values.

### 2.2 Freeze–thaw lyophilised and ensilicated lysozyme

Lyophilised and ensilicated lysozyme was subjected to the following freeze–thaw conditions: 1 hour freeze–thaw cycle at −20 °C, total of 3 cycles; 24 hours freeze–thaw cycle at −20 °C, total of 1 cycle; 2 months frozen–thawed at −20 °C; 3 months frozen–thawed at −20 °C. Samples were reconstituted or released and dialysed in the respective buffer required for analysis either PBS or 50 mM Tris pH 7.

### 2.3 Field emission scanning electron microscopy (FE-SEM) of ensilicated lysozyme

Ensilicated material in powdered form was gently ground for deposition onto a metal stub containing carbon tape. The samples were vacuum dried overnight before imaging using the JEOL FESEM6301F at 20 000× magnification.

### 2.4 Differential scanning calorimetry (DSC) of native, released and ensilicated lysozyme

#### 2.4.1 Native and released lysozyme

Microcalorimetry (Setaram, MicroSC multicell calorimeter) was performed on native and released lysozyme. Native lysozyme (10 mg ml^−1^) and released lysozyme (∼4 mg ml^−1^) were analysed, at a volume of 0.5 ml, using Hastelloy C276 closed cells with PBS buffer as reference at the same volume. Samples were held at 20 °C for 5 minutes before undergoing 1 cycle from 20–95 °C at a rate of 1°C min^−1^. The experiments were run under nitrogen gas sweeping. Triplicate runs were done to ensure validity of the results and a buffer blank run was included as a control to eliminate buffer interactions.

#### 2.4.2 Ensilicated lysozyme (heating)

Ensilicated lysozyme powder (5–7 mg) was added to an aluminium calorimetry pan. The DSC apparatus (DSC Q20, TA instruments) was set to run a temperature range from −10C° to 100 °C for 3 cycles at 5 °C min^−1^ followed by a hold at each extremity for 5 min. After the cycles were complete, material was held at 100 °C for 5 hours to assess thermal resilience. Fourier-transform infrared (FT-IR) spectra were collected for each sample before and after it underwent DSC to detect any changes in structure as a result of heating. Ensilicated lysozyme powder (10 mg) was also analysed by microcalorimetry with air as a reference, using the same temperature range as detailed above.

### 2.5 Thermogravimetric analysis – differential thermal analysis – mass spectrometry (TGA-DTA-MS) of ensilicated and lyophilised lysozyme

Thermogravimetric (TGA) differential thermal analysis (DTA) coupled with mass spectrometer (MS) was performed on lyophilised and ensilicated lysozyme material. A Setsys Evolution 16/18 (Setaram) was used with alumina pans as sample holders; the reference pan was left empty. The analysis, where 5.9 mg (ensilicated) and 7.2 mg (lyophilised) lysozyme was used, was run from 30 °C to 200 °C at 10 °C min^−1^ under argon flow at 20 mL min^−1^. Alongside with the DTA signal, the mass evolution of the sample was measured by thermogravimetry (TGA) and evolved gases were detected using a mass spectrometer (MS) attached to the instrument (Omnistar GSD 320, Pfeiffer Vacuum, equipped with a quadrupole mass analyser and a SEM detector) through a stainless steel capillary. The MS was set to record atomic mass units (amu) from 1–200 for the duration of the run. The known decomposition elements for proteins were investigated for (increasing) presence during the analysis.

### 2.6 Circular dichroism

#### 2.6.1 Native *vs.* released lysozyme

CD was utilised to measure protein unfolding of ensilicated lysozyme at standard (1 : 50) conditions compared to reconstituted lyophilised lysozyme. Spectra were measured between 260–185 nm for a temperature range of 60–80 °C at 1 °C min^−1^ increments. Normalised sample absorbance at 222 nm was integrated with a thermodynamic fit and elucidated *T*_m_ values for each condition, respectively. Samples were buffer-exchanged to 10 mM sodium phosphate buffer at pH 7 for measurement.

#### 2.6.2 Freeze–thaw

Reconstituted and released lysozyme exposed to freeze–thaw conditions were analysed by measurement of CD spectra between 260–185 nm. Freshly reconstituted lysozyme was used as reference control. Measurement values were converted to delta epsilon (Δ*ε*) for normalisation.

### 2.7 Long-term stability ensilicated lysozyme *in vivo*

Lyophilised lysozyme (native control) was dissolved in 50 mM Tris–HCl pH 7 buffer and heat-treated at 95 °C for 5 hours (−ve control).

#### 2.7.1 Sample preparation before intra-peritoneal injection

Ensilicated samples were released in 1 : 1 ratio of 190 mM NaF pH 2.65 and Tris–HCl pH 7 buffer. Ensilicated lysozyme (7.5 mg), ensilicated heated treated lysozyme (7.8 mg) and ensilicated lysozyme (5.8 mg, master sample for comparison) were dissolved in 10 ml of release buffer. After 1 hour incubation on a roller bank, 3 ml of each sample was added to a Slide-A-Lyzer™ 10k MWCO dialysis cassette to be buffer-exchange into Tris–HCl overnight at 4 °C. The following day, samples were extracted from their cassettes. Quick UV-absorbance analysis using a NanoDrop™ One Microvolume UV-Vis Spectrophotometer (Thermo Scientific) analyser verified protein quantity. Samples were filtered using a 0.22 μm spin column. Samples were sterile filtered for 1 min at 16 000 × *g*. Final concentrations were checked using the NanoDrop system. 50 μg of lysozyme, with 10 μg of LPS (Sigma, UK, #L4391-1MG, LPS from *E. coli* 0111:B4, stock reconstituted in sterile saline buffer to 1 mg ml^−1^) added, was used per dosage.

#### 2.7.2 Animal mouse study

The lysozyme *in vivo* study included 15 mice with 5 mice designated per group under the following conditions: native denatured (−ve), ensilicated released and ensilicated master sample (long term storage > 3 years), released. Each mouse received an intra-peritoneal injection. Blood was collected by tail venesection on days 0, 7, 14, 21, 28, 35 with a final bleed on day 42. At day 28, mice were given a booster dose from a stored sample of the same condition.

#### 2.7.3 ELISA serum analysis

Native lysozyme was coated on a 96-well microtiter plate (10 μg ml^−1^, 100 μl per well) at 4 °C overnight in PBS pH 7.4. Plates washed 3× with PBS and blocked with 1% Casein in PBS-Tween 20 (0.05%) for 2 hours. After four washes serum was added in a two-fold serial dilution starting at 50 times dilution. Monoclonal, Hyhel-10,^32^ antibody was added to four wells in each plate to normalise variation across microplates. After 1 hour incubation, goat-anti-mouse IgG + HRP was added in 1 : 5000 dilution to PBS-T (0.05%) and added to each plate for another 1 hour incubation at room temperature. Finally, after extensive washing, TMB substrate was added and the reaction ended after addition of 10% H_2_SO_4_. Absorbances were read at 450 nm with a reference at 650 nm and subtracted subsequently.

#### 2.7.4 ELISA data processing

Serum absorbances collected for all conditions were normalised using the HyHel-10 monoclonal antibody responses and corrected for serum volumes. These values were output in relative units (RU) and allowed comparison between all ELISA plates. Samples (*n* = 5 per group) were plotted in RU responses at day 42.

### 2.8 EnzCheck™ enzyme activity analysis

Enzyme kinetic analysis (EnzCheck™ lysozyme assay kit, ThermoFisher, UK), normalised using protein concentration determined by BCA assay, validated the samples for use in the *in vivo* study. The analysis was performed according to manufacturer's instruction where fluorescence intensity (excitation 485 nm and emission at 520 nm) was measured. Sample values within the standard curve were interpolated and are presented as units per mg of protein.

## 3 Results & discussion

### 3.1 Thermal resilience of ensilicated lysozyme against heating with varying silica ratios

**Fig. 1:**
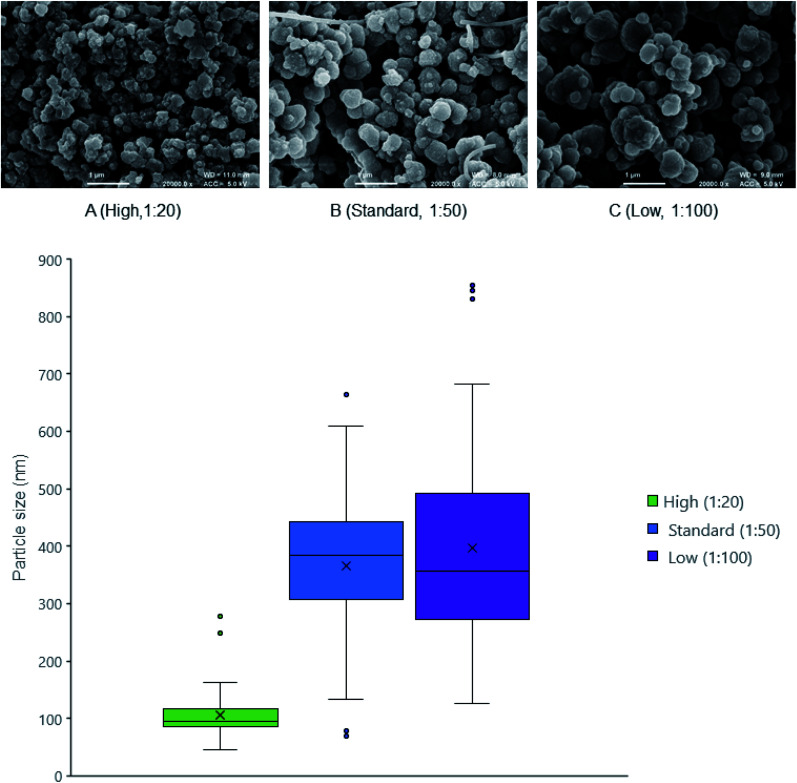
FE-SEM of ensilicated lysozyme. (A) 1 : 20 ensilicated lysozyme (B) 1 : 50 ensilicated lysozyme (C) 1 : 100 ensilicated lysozyme. Ratios correspond to the volume of pre-hydrolysed TEOS added to bulk reaction volume. Images taken at 20 000× magnification scale bar represents 1 μm. Particle size distribution (bottom) shows distribution of measured diameters using ImageJ.

An earlier study into the mechanism of ensilication showed a diffusion limited (controlled) cluster aggregation (DLCA) type process.^10^ This means the more silica added to the reaction, the faster the ensilication will occur because of the abundance of polymeric silica.^33,34^ This was observed by FE-SEM (Fig. 1 and Table 1) when lysozyme was ensilicated at various ratios. The nanoparticles at high silica ratio became stabilised earlier. Observing the ratios from high to low silica led to formation of smooth larger particles showing decreased agglomeration.

**Table 1:** Ensilication process at various silica to reaction volume ratios^a^

Ensilication	High	Standard	Low
Visual turbidity	Fast (seconds)	Normal (minutes)	Slow (tens of minutes)
Ratio silica : volume	1 : 20	1 : 50	1 : 100
Particle size (d.nm)	95 ± 40	380 ± 130	360 ± 180

a Particle growth indicates the visible occurrence of turbidity. Ratio defines the volume amount of hydrolysed TEOS added to protein solution. Particle size is measured using ImageJ and displayed as median with standard deviation.

To understand the stabilising effect with various levels of ensilication, we utilised DSC to probe the thermal resilience. Control experiments (*n* = 3) were performed with native lysozyme in PBS, to establish protein unfolding as an endothermic transition and to calibrate our experimental setup. Results revealed good consistency with literature values, which report lysozyme to have an unfolding temperature at 72.9 °C.^24,35–37^

**Fig. 2:**
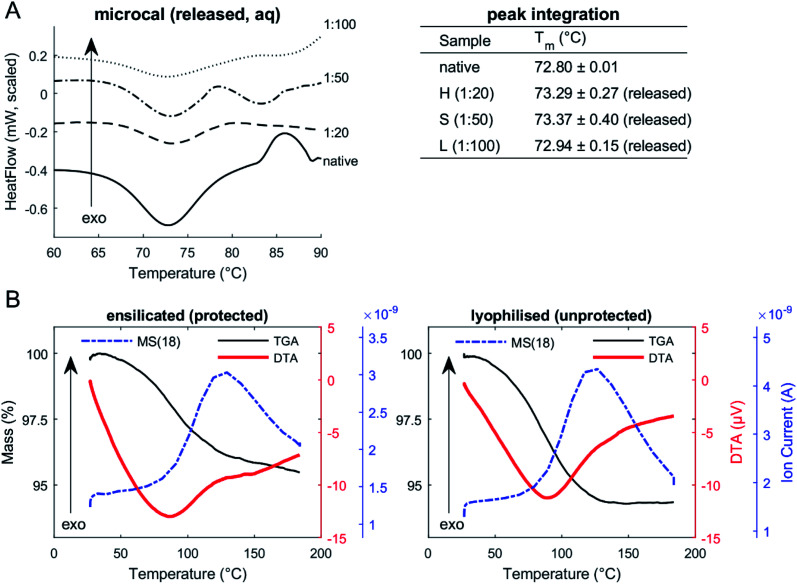
(A) Micro-DSC of lysozyme (in solution). Native and released lysozyme in PBS. Peak integration of endothermic transitions confirms (peak) *T*_m_ of both native and released to be around 72.9 °C. (B) TGA-DTA-MS of ensilicated (protected) and lyophilised (unprotected) lysozyme (powder). TGA of ensilicated and lyophilised material displays an average 5% weight reduction. Mass spectrometer signal (dashed-dotted line) for atomic mass unit 18: MS(18), water, is apparent through heating.

DSC of released protein from ensilicated material (Fig. 2, Table 2) displayed similar *T*_m_ to native lysozyme. We also found these values when performing an unfolding ramp using CD of (released) ensilicated (1 : 50 ratio) lysozyme confirming our observations (Fig. S1†) with the *T*_m_ for native found at 73.47 ± 0.12 °C and released at 72.10 ± 0.99 °C.^31^

**Table 2:** Microcalorimetry (μDSC) and circular dichroism (CD) unfolding temperatures of lysozyme^a^

*T*_m_ of lysozyme *via*	μDSC	CD
Native	72.80 ± 0.01 °C	73.47 ± 0.12 °C

**Ensilicated (released)**		
High (1 : 20)	73.29 ± 0.27 °C	—
Standard (1 : 50)	73.37 ± 0.40 °C	72.10 ± 0.99 °C
Low (1 : 100)	72.94 ± 0.15 °C	—

a Both methods measured thermodynamic transitions of lysozyme undergoing a temperature ramp. Heat-flow and far-UV circular dichroism was analysed and the *T*_m_ obtained by mathematical fitting (*n* = 3).

**Fig. 3:**
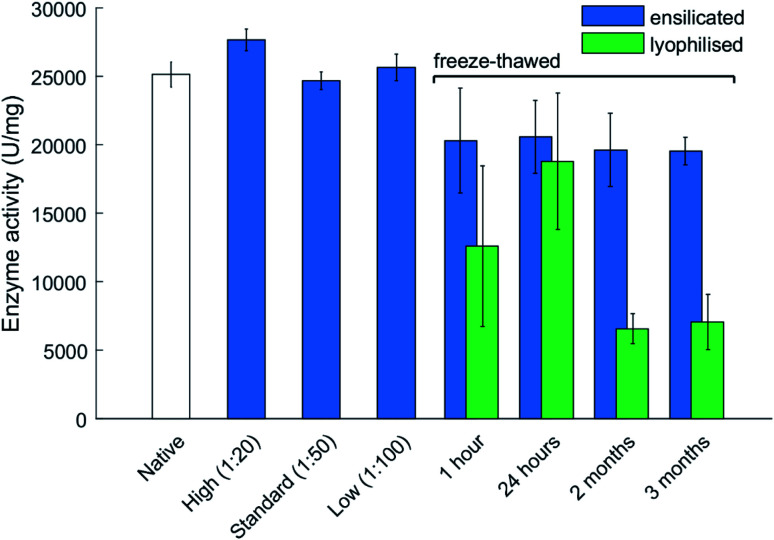
Enzyme activity at various ratios of ensilication and after freeze thawing. The freeze–thaw stability assessment is presented for various durations of time. Ensilicated and lyophilised material were exposed to storage at −20 °C for 4 time points. Material was reconstituted/released after each and its activity measured. Data represents activity for each sample with error bars representing standard deviation (*n* = 3). Samples all normalized for protein concentration using the BCA assay. Ensilicated material used for the freeze–thaw assessment is at 1 : 50 ratio. Ratios correspond to the volume of pre-hydrolysed TEOS added to bulk reaction volume. The 1 hour freeze–thaw data displays enzyme activity after 3 cycles of 1 hour freeze–thaw.

Enzymatic functional assessment of released lysozyme, where ensilicated form was used in calorimetric analysis (Fig. 3), revealed no alterations in their functionality and are in alignment with previously published results.^8^ To support the powder DSC results, TGA-DTA-MS was utilised to confirm that the mass loss of (1 : 50 ratio) ensilicated material during heating was associated with free water liberation (Fig. 2). On average, 4.6% (*n* = 2) loss of mass was measured. An additional control was performed where ensilicated lysozyme was compared to lyophilised material with an average 6.5% (*n* = 3) loss of mass during the experiment. This was accompanied by an endothermic DTA peak which is typically in line with water evaporation. The results displayed no differences between lyophilised and ensilicated lysozyme. The mass spectrometry data showed an increase at amu 18, representative of H_2_O molecules, as the mass of sample decreased in the 30–200 °C heating window. This validated our findings that it was H_2_O.

Our analysis suggests that there was no observable effect of ensilication to the protein that would cause a change in the vibrational energy required for it to denature. This confirms our earlier understanding that the interaction between silica and protein is purely electrostatic. In addition, powder DSC analysis (Fig. S2†) on the three ensilicated lysozyme batches revealed no transitions that could indicate protein unfolding. Only during the first cycle (total of 5) of stress testing, an endothermic slope was observed. We believe this to be due to forced liberation of attached residual water in the material after it was vacuum dried. After the first cycle of thermal stress, no further transitions were identified. FT-IR analysis of ensilicated material before and after stress test verified our findings (Fig. S3†), displaying an overall increase in IR transmission at all hydrogen and oxygen containing wavenumbers. Silicon (^29^Si) NMR showed no presence of silica after release (Fig. S4†).

The TGA-DTA-MS result demonstrates that ensilicated material possesses a similar thermal resilience to the lyophilised material. This is either due to the silica coat absorbing the thermal energy, the physical immobilisation preventing protein unfolding or a combination of both. This could shift the endothermic transition towards a high *T*_m_, but this is beyond the scope of this study.

### 3.2 The effect of freezing on ensilicated lysozyme at standard conditions

The main advantage of ensilication is that ensilicated material does neither require refrigeration or desiccation for storage and transportation. However, given the substantial amount of water in the ensilicated material, the subsequent step of this study was to focus on the thermal resilience against freezing. Additionally, it would be supplementary to understand how ensilicated material would protect in colder climates.

Freezing of proteins weakens inherit hydrophobic interactions and can alter protein structure after thawing. Experiments where ensilicated and lyophilised lysozyme samples (*n* = 3 for each time point and category) were frozen revealed ensilication to provide a consistent amount of functional retention. The catalytic activity of lyophilised material significantly dropped after being frozen for 2 months at −20 °C then thawed (Fig. 3). This decrease was not observed for ensilicated material suggesting improved retention of ensilicated lysozyme and superior thermal resistance compared to lyophilised proteins.

From the TGA-DTA-MS data, it is evident there is some H_2_O present after ensilication. This amount does not appear to affect the protein after freezing and provides more evidence on the conformational restriction the silica shell inflicts on the embedded proteins. CD analysis of the 3 month samples showed slight unfolded spectra for lyophilised and ensilicated material compared to freshly reconstituted lysozyme (Fig. S5†). This result indicates some loss in secondary structures. However, enzymatic activity was retained. This was reflected in the analysis and supplemented our findings (Fig. 3).

### 3.3 The long-term antigenic stability of ensilicated (released) lysozyme injected in mice

**Fig. 4:**
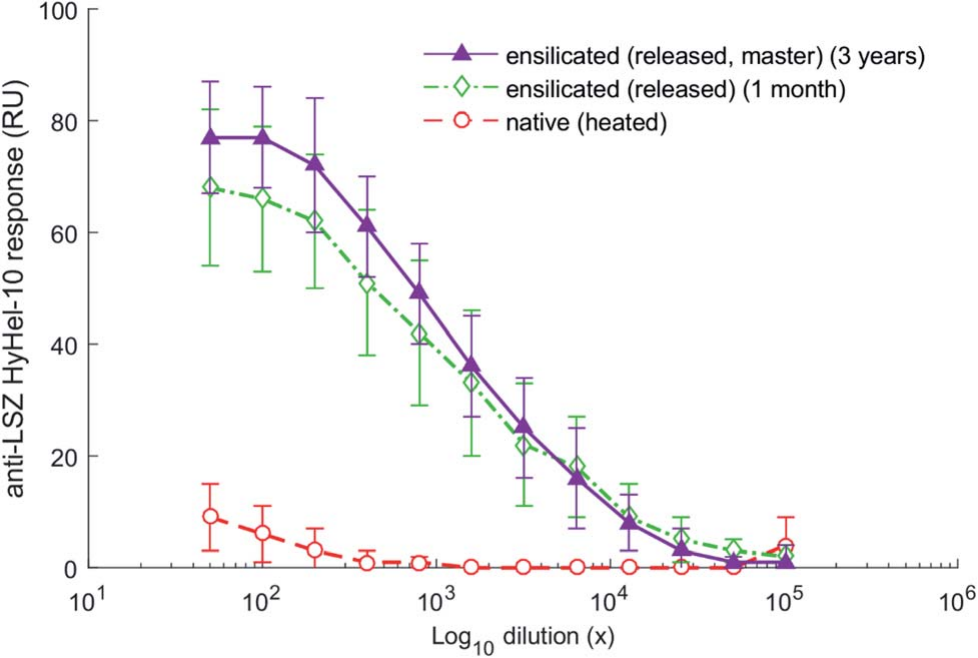
ELISA serum responses at day 42 of the *in vivo* mice trial. Responses (*n* = 5 per group) displayed in relative units (RU) to the HyHel-10 monoclonal antibody against HEWL. ANOVA with *post hoc* Tukey shows statistically significant difference between ensilicated samples and native heated (*P* < 0.01). No significant difference was calculated for the comparison between ensilicated materials (*P* > 0.05).

As a commonly used antigen, HEWL is able to elicit an immune response when injected into mice. The *in vivo* experiment used ensilicated lysozyme samples made at standard (1 : 50) condition stored for 1 month and 3 years. Significant responses to HEWL were not detectable until one week after boost in all cases with the data from the final bleed at day 42 (Fig. 4) being most representative. We observed good distinction between native-heated and both ensilicated-then-released protein (*P* < 0.01, *n* = 5). The data clearly indicates that long-term storage of ensilicated lysozyme at ambient temperature does not impair protein stability. Additionally, enzyme activity analysis to assess enzyme function showed similar results between native (31 747 U mg^−1^) and ensilicated materials, 34 533 and 29 826 U mg^−1^ for 1 month and 3 years released sample respectively, where native-heated lost all functionality (66 U mg^−1^) which supports our findings.

## 4 Conclusion

Our previous studies have provided the proof-of-principle that ensilication protects proteins from thermal degradation, and subsequently elucidated the stabilisation mechanism. Here, we set out to assess the thermal resilience of ensilication with use of calorimetric methods and *in vivo* analysis. Our results demonstrate that the protein is unaffected when ensilicated even after exposure to harsh high (denaturing) temperatures, where native unprotected proteins unfold and denature.

The ensilicated material contains bound water molecules that are liberated under elevated thermal stress, but there were no observed transitions that could indicate protein degradation/unfolding within the material. This suggests rigid confined protection of protein structure and/or absorption of thermal energy *via* the silica shell. Silica ratios even at low amounts (1 : 100) seem to protect against thermal denaturation for lysozyme. The evidence presented in this study depicts ensilication to provide a high level of protection against thermal degradation that proteins normally would undergo in solution. Enzymatic analysis confirmed retention of functionality. In contrast, ensilicated lysozyme subjected to freeze-thawing retained its activity up to 3 months. It suggests humidity does not influence ensilicated material, in contrast to some other preservation methods such as sugar glass encapsulation.

In conclusion, ensilicated lysozyme is thermally resilient against freezing and heating which after release has retained its native properties. Long-term storage is feasible up to 3 years at ambient temperature and promises a significant decrease in costs associated with current cold chain logistics. When compared to lyophilisation, this methodology has similar thermal resistance if exposed to heating, indicating that any therapeutics that cannot withstand lyophilisation, could instead be ensilicated for long-term storage and easier transport without refrigeration. In turn, this would remove cold chain logistics for transport of therapeutics, reducing the cost of transport and storage, removing the need for refrigerator equipment, dependence on electricity and infrastructure, and reducing the waste from degraded biopharmaceutical products.

## Author contributions

A. S. conceived the study. A. S. and A. D. designed the experiments. A. D., L. C., M. N. S. and L. W. performed the experiments. R. C. provided experimental and academic assistance with the calorimetric analysis. Y.-C. C provided the long-term storage ensilicated samples. J. P. performed the *in vivo* trial under supervision of K. J. M. F. K., K. J. E. and J. vd. E. provided academic support. A. D., L. C. and M. N. S. wrote the manuscript with contributions from all authors.

## Ethical statement

Mice were housed at the comparative biology centre, Newcastle University; all experiments were conducted in accordance with institutional guidelines, approved by AWERB and by the Home Office of the United Kingdom, under the auspices of project licence P35D9C60C.

## Funding

A. D. thanks the Annett Trust and University of Bath for funding. M. N. S., L. C. and L. W. thank the University of Bath. M. N. S. additionally thanks Roger and Sue Whorrod for funding. FK is funded by the Medical Research Council (MR/P002927/1). A. S. thanks the Royal Society and University of Bath for funding. K. J. M. and J. P. were funded by an MRC CiC grant from Newcastle University.

## Conflicts of interest

The authors declare that the research was conducted in the absence of any commercial or financial relationships that could be construed as a potential conflict of interest.

## Supplementary Material

RA-010-D0RA06412B-s001
