# Supplementary material for: Thermal resilience of ensilicated lysozyme via calorimetric and in vivo analysis
Source: RSC Adv. 2020 Aug 12;10(50):29789–96. doi: 10.1039/d0ra06412b (PMC9056174; doi:10.1039/d0ra06412b)
Supplement: RA-010-D0RA06412B-s001 [file RA-010-D0RA06412B-s001.pdf]

Electronic Supplementary Material (ESI) for RSC Advances.
This journal is © The Royal Society of Chemistry 2020

Supplementary - Thermal resilience of ensilicated lysozyme via calorimetric and *in vivo* analysis.

A. Doekhie[1], M. N. Slade[1], L. Cliff[1], L. Weaver[1], R. Castaing[2], J. Paulin[3], Y-C. Chen[1], K. J. Edler[1], F. Koumanov[4], K.J. Marchbank[3], J.M.H van den Elsen[5] and A. Sartbaeva*[1].

[1]Department of Chemistry, [2]Material and Chemical Characterisation Facility, [4]Department of Health, [5]Department of Biology and Biochemistry. University of Bath, Claverton Down, Bath, BA2 7AY, United Kingdom.

[3]The Medical School, Framlington Place, Newcastle University, Newcastle upon Tyne, NE2 4HH, UK.

Supplementary - Thermal resilience of ensilicated lysozyme via calorimetric and *in vivo* analysis.

CD calorimetry native vs released 1:50

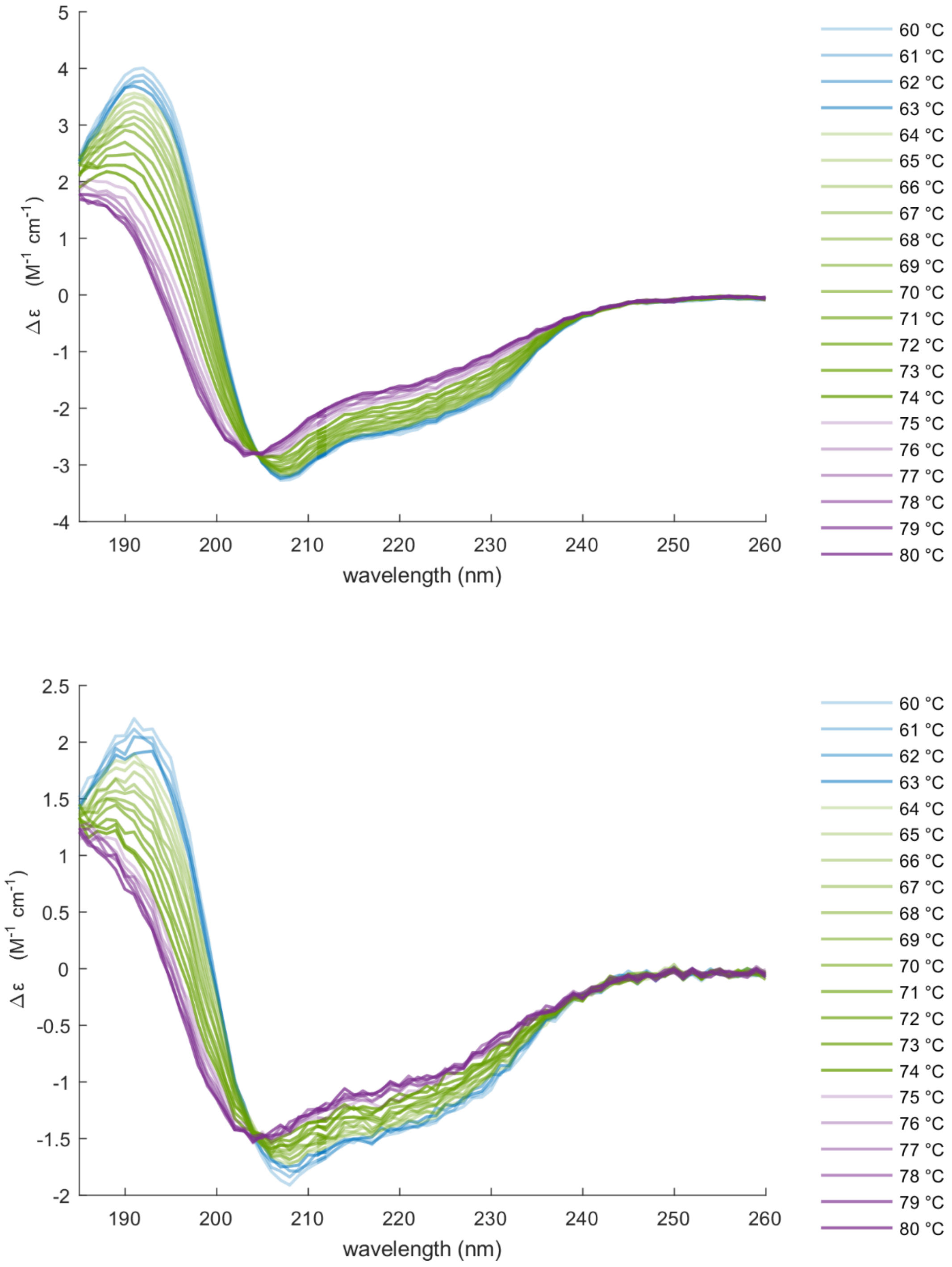

Figure S1A. CD spectra of lysozyme over thermal ramp. (top) native lysozyme, (bottom) released lysozyme. Delta epsilon units represent normalised data.

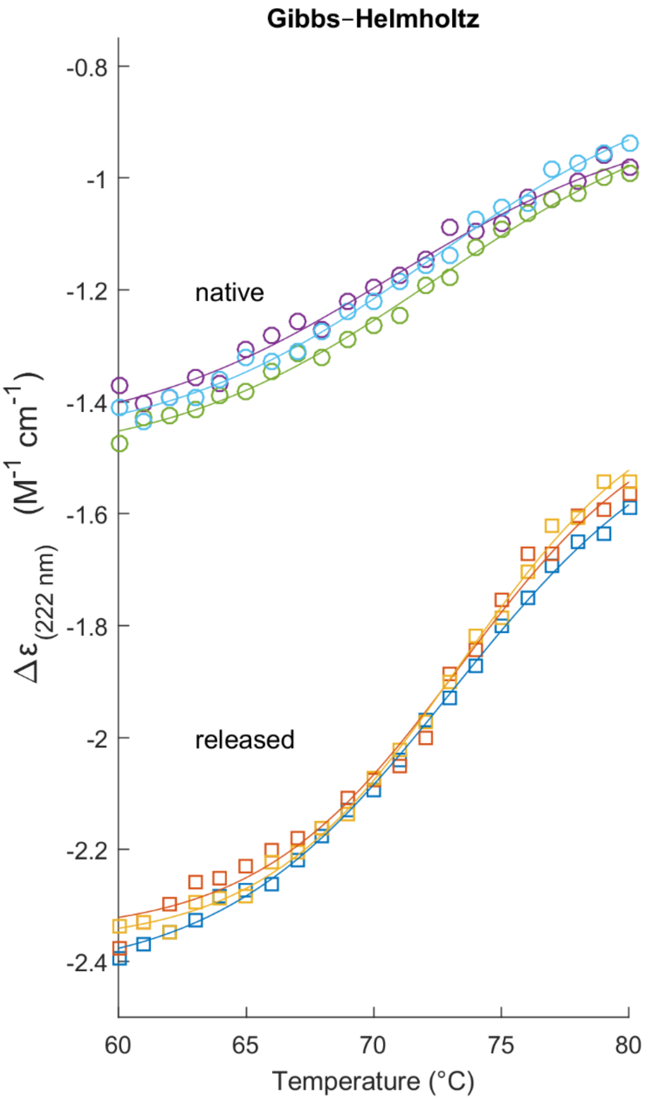

Figure S1B. Modified Gibbs-Helmholtz thermodynamic fit to delta epsilon values at 222 nm for native and released lysozyme (n=3). T_m for native was found at 73.47 ± 0.12 °C and released 72.10 C ± 0.99 °C (n=3 for both samples).

Powder DSC

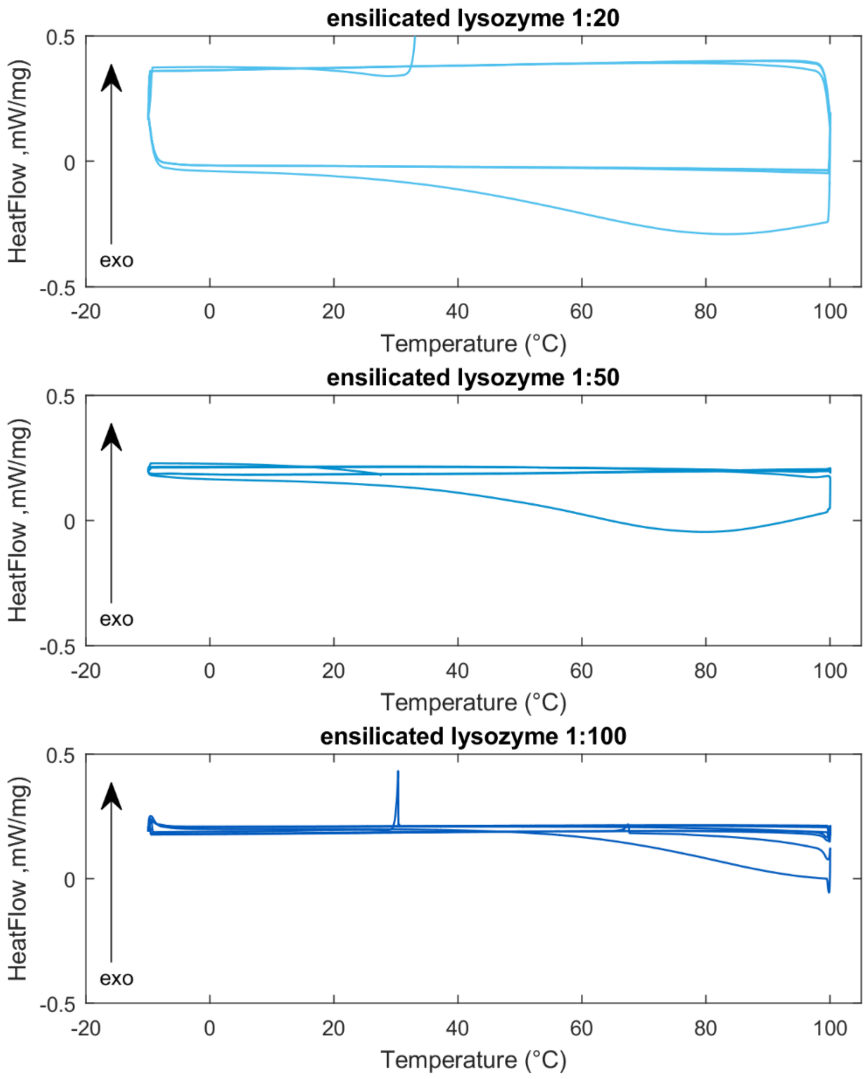

Fig. S2. Powder DSC of ensilicated lysozyme at 1:20/1:50/1:100 ratio of ensilication. DSC runs of ensilicated lysozyme at various ratios display similar signals. Initial ramp shows an endothermic slope, as there is a broad endothermic dip present. . This signal disappears after the first cycle. No further transitions observed indicating the absence of physiochemical changes and suggest protein inside the material being protected and is verified using TGA-DTA-MS.

FT-IR

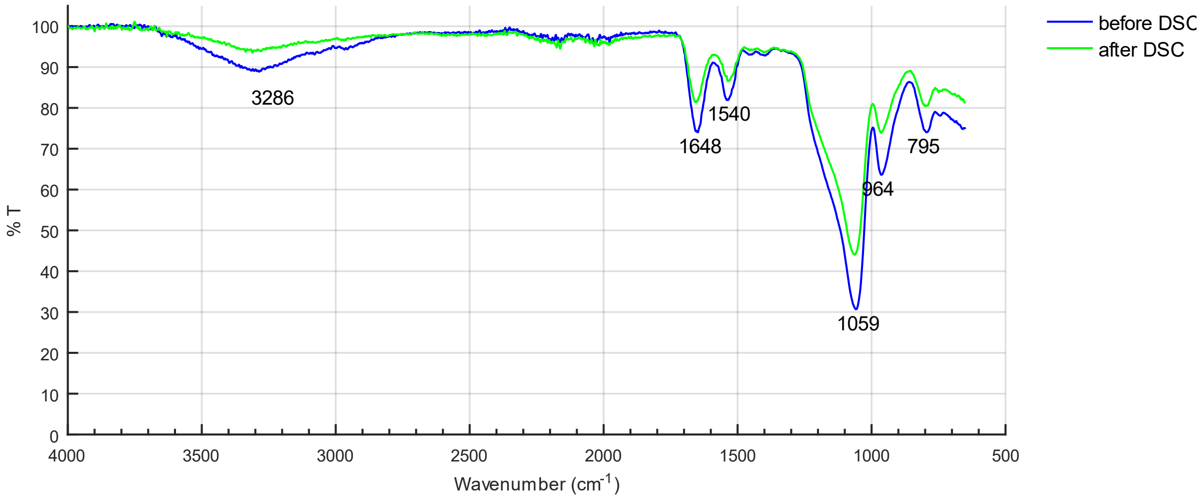

Figure S3. FT-IR of ensilicated lysozyme. Ensilicated lysozyme (1:50) before and after thermal stress testing using DSC. The FT-IR shows an increase in % transmission after the thermal run. There is an overall reduction in absorbance of bonds associated with O-H (3286/1648/1059 cm^{-1}) and -H (1540 cm^{-1}).

Silicon (^{29}Si) NMR

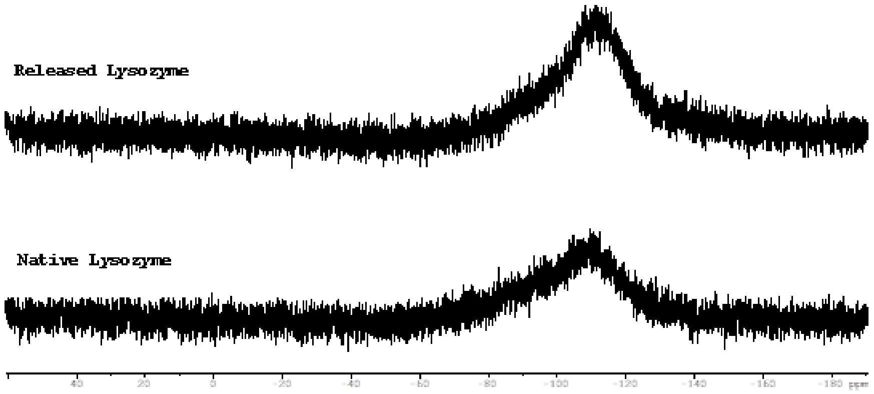

Compound	Chemical shift (ppm)
SiF_4	-112
SiO_2	-107
H_2SiO_3	-102
H_2SiF_6	-119

Figure S4. (top) Silicon (^{29}Si) NMR of released and native lysozyme. Range is set between +60 and -200 for detection of relevant silicon containing silica species[1] (table) . Native lysozyme spectra showed a peak due to the glass sample holder. The small shift in released sample spectra was found not to be significant and confirms the absence of silica present after release and dialysis.

CD of 3 months frozen HEWL and ensilicated material

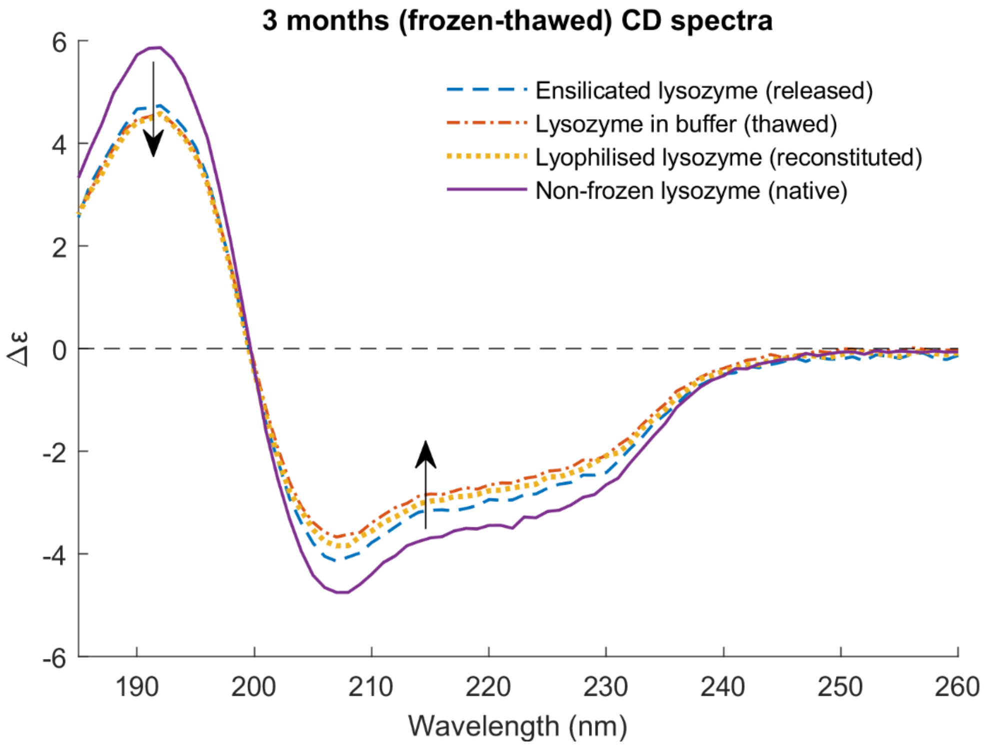

Figure S5. CD spectra of freeze-thawed samples after 3 months storage at -20 °C. Non-frozen lysozyme is control sample. Lyophilised lysozyme material was stored as powder and after thaw reconstituted. Lysozyme was also stored in buffer solution and thawed. Ensilicated sample was released after thaw. All samples were prepared in 10 mM sodium phosphate buffer at pH 7 before analysis. All samples were background corrected and normalised to delta epsilon based on the concentrations obtained via BCA protein assay. There is a decrease in chirality observed for all samples except control indicated by black arrows.

References

1 Uhlig, F. & Marsmann, H. C. ^{29}Si NMR Some Practical Aspects. 208-222 (Dortmund University Paderborn University, Germany, 2000).
